# Evaluating the clinical application of a leaflet for clinical practice guideline in patients with lumbar herniated intervertebral discs: Randomized controlled trial

**DOI:** 10.1097/MD.0000000000009406

**Published:** 2017-12-22

**Authors:** Ju Ah Lee, In-Hyuk Ha, Tae-Young Choi, Jiae Choi, Ji Hee Jun, Byoung-Kab Kang, Myeong Soo Lee

**Affiliations:** a KM Fundamental Research Division, Korea Institute of Oriental Medicine, Daejeon; b Jaseng Spine and Joint Research Institute, Jaseng Medical Foundation, Seoul, Republic of Korea; c Clinical Research Division, Korea Institute of Oriental Medicine, Daejeon; d Department of Korean Internal Medicine, College of Korean Medicine, Gachon University, South Korea.

**Keywords:** clinical practice guideline, clinical research protocol, effect, herniated lumbar disc, leaflet, traditional Korean medicine

## Abstract

**Background:**

This study aims to demonstrate the effectiveness of using clinical practice guideline (CPG) leaflets as a communication tool between doctors and patients. We evaluated the leaflets as a communication tool in the treatment of lumbar herniated intervertebral discs (HIVDs) in terms of patient and physician satisfaction and ease of treatment.

**Methods:**

This study is a 2-parallel-arm, assessor-blinded, randomized controlled trial at Jaseng Hospital, Seoul, South Korea. We evaluated efficacy through a comparison of satisfaction and clinical outcomes in randomly allocated groups of HIVD lumbar patients visiting Jaseng Hospital of Korean Medicine. We used leaflets on the basis of Korean medicine CPG recommendations as an intervention. The intervention group received treatment and diagnosis using the leaflet, and the control group received the typical intervention, which was provided without the leaflet.

**Results:**

The levels of patient satisfaction with and understanding of the doctors’ explanation was 92% in the leaflet group and 64% in the nonleaflet group, which showed that, compared with patient satisfaction in the nonleaflet group, patient satisfaction was considerably higher by 28% in the leaflet group. In addition, the level of the reliability with treatment was 92% in the leaflet group and 64% in the control group. However, there were no significant differences in statistical analyses. The level of doctors’ satisfaction with communicating with patients using the leaflet was 100% in the leaflet group. Given this satisfaction, the ease of persuasion of treatment was highest (84%) and followed the improvement in the patient's understanding of the treatment (16%) in the leaflet group. In addition, in the nonleaflet user group, almost all doctors thought that having a leaflet would be a more effective treatment.

**Conclusion:**

Although this study failed to show significant differences between the intervention and control groups, the leaflet, which included CPG information in the treatment of HIVD patients, was an effective communication tool between patients and doctors. However, further studies with larger samples should be conducted to investigate the effectiveness of the communication tool based on the CPG.

Trial registration number: Clinical Research Information Service: KCT0001762

## Abstract 2

Supplemental Digital Content is available in the text

## 1 Introduction

Lumbar herniated intervertebral disc (HIVD) is a disease caused when damage to the discs and the soft gel inside them pushes through their walls and presses against the nerves or the spinal cord, causing a burning pain in the legs and pain in the back.^[1]^ HIVD is a very common disease, with a reported lifetime occurrence as high as 40%.^[2]^ In a large cohort of 19-year-old Korean males, the prevalence of adolescent HIVD was 0.60%.^[3]^ In addition, Korean medicine (KM) treatments are predominantly used for musculoskeletal disorders, including HIVD.^[4]^

However, the treatment processes are diverse, and the proper management of evidence-based guidelines is needed in Korea.^[5]^ To address this issue, clinical practice guidelines (CPGs) have been developed with traditional medicine in Korea.^[6]^ Despite the existence of CPGs, the application of recommendations into routine clinical practice remains very slow.^[7]^ However, the utility of CPGs is more important in developing its widespread use.

Recently, several studies have examined the implementation of evidence or guidelines.^[7–9]^ Among several methods for the implementation of CPGs, there are clinical studies that have demonstrated the applicability of CPGs to clinical fields.^[8,10–13]^

In the current study, we aimed to investigate the effectiveness of CPGs as an implementation and communication tool. We created leaflets describing evidence-based CPGs. Then, respondents were divided into 2 groups: a group in which respondents did not use a leaflet (the nonleaflet group) and a group in which they did (the leaflet group). Thus, the applicability of evidence-based CPGs was evaluated. The primary objective of this study evaluated the effectiveness of the leaflet as a communication tool between patients and doctors.

## 2 Methods and design

### 2.1 Study design

**Figure 1:**
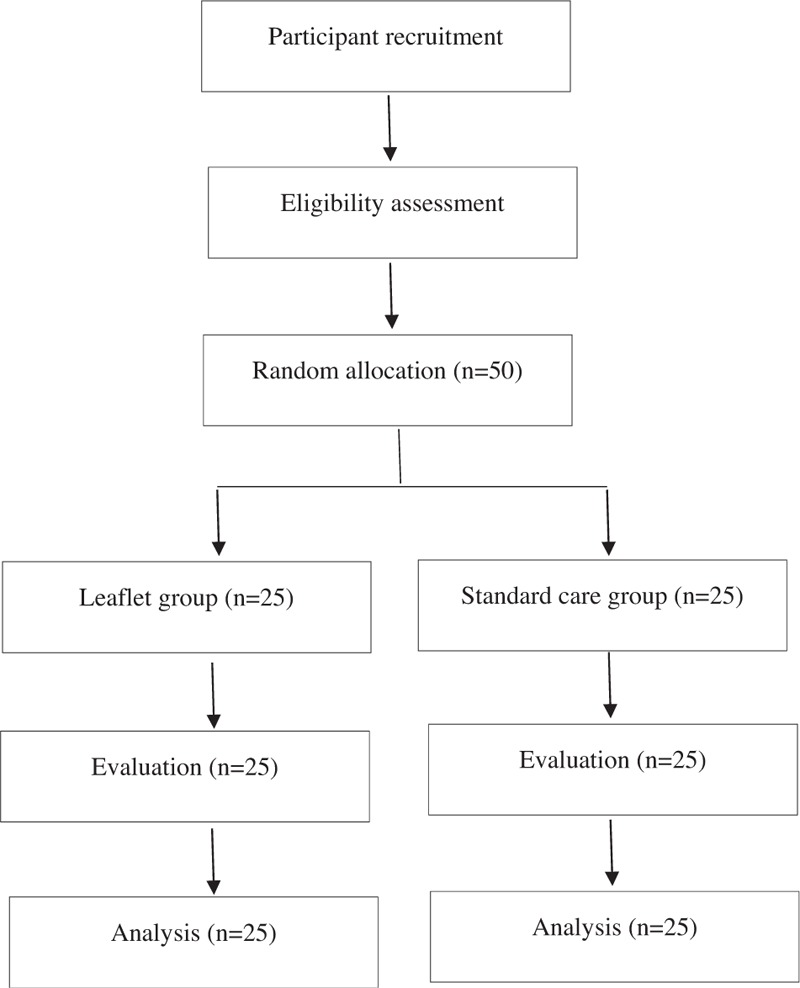
A flowchart of the study.

This study is a randomized controlled trial with 2 parallel arms and an assessor-blinded design. The trial was performed at the Jaseng Korean Medicine Hospital in Korea in accordance with the Declaration of Helsinki and the Guidelines for Good Clinical Practice. The protocol of this study has been registered with the Clinical Research Information Service (CRIS), which is the Korean registry of the World Health Organization Registry Network. We also published the study protocol in a peer-reviewed journal.^[14]^ Eligible participants were randomly divided into either the leaflet group or to the standard care group, with a 1:1 allocation ratio (Fig. 1). The evaluation of participants and the analysis of the results were performed by professionals blinded to the group allocation.

### 2.2 Study participants

A total of 50 patients were recruited through local advertising and from outpatients at the Jaseng Korean Medicine Hospital. The inclusion criteria were patients of both genders between the ages of 18 and 65 years who were diagnosed with HIVD using computer tomography (CT) or magnetic resonance imaging (MRI). All patients provided informed written consent to participate and agreed to comply with the study regulations.

The exclusion criteria included heart disease, liver disease, kidney disease, any psychiatric condition, the inability to communicate, critical illness, pregnancy, or any condition that could influence the study assessment.

### 2.3 Randomization

The study participants who met the eligibility criteria were randomly assigned to 1 of 2 groups (the leaflet group or the standard care group) at the first visit using a central randomization system with a 1:1 ratio. Randomization was conducted with a computer-generated random allocation sequence using the stratified block randomization method of the SAS package (version 9.1.3; SAS Institute, Inc., Cary, NC) and was performed by a statistician with no clinical involvement in this trial. The size of the block was 2. The allocation concealment was ensured because the randomization code was only released after the participants were recruited to the trial and after all baseline measurements are taken. The subjects and practitioners were aware of the allocation given the routine care setting. However, the outcome assessors and the statistician performing the data analyses were blinded to the treatment allocation.

### 2.4 Blinding

The outcome assessor was blinded, but the participants and doctors could not be blinded.

### 2.5 Interventions

#### 2.5.1 Leaflets based on CPGs

The leaflet group received an explanation of the overall treatment and diagnosis of HIVD based on a leaflet that included recommendations and was evidence-based on traditional KM (TKM) CPGs. This leaflet was created to improve the communication between doctors and patients and to provide information to both groups (Supplementary Figure 1). Clinicians participating in the study generally agree with the guidelines’ recommendations and content. In addition, the participants in the study were pre-educated so that they would not be influenced by their usual thinking, and were taught that the other treatments were almost identical, except for leaflet-based explanations.

#### 2.5.2 Standard explanation of diagnosis and therapy

The patients in this group received general information regarding the diagnosis and treatment of HIVD. We conducted standard operation procedure (SOP) training for every practitioner to unify the process; this training included the following: general diagnosis and method for prognosis and administration of the TKM intervention and other treatments following prognosis.

### 2.6 Data collection

The data were collected to develop a leaflet describing evidence-based CPGs that were prepared in collaboration with TKM hospitals. Through the analysis of outcome measures, we reviewed the pattern of medical treatments derived from key recommendations based on these evidence-based CPGs. In addition, we prepared a leaflet describing evidence-based CPGs regarding herniation of the lumbar vertebrae. Thus, we evaluated the applicability of evidence-based CPGs in an actual clinical setting. All data were collected and analyzed without the inclusion of any identifying information.

The outcome measurements were verified by an independent assessor for each patient. These data were entered into the case report form by a certificated clinical research coordinator.

When we received informed consent from patients, we informed patients as follows: “This study seeks to assist in treatment decision-making by utilising a leaflet, which describes the CPGs, as a tool for communication between patients with herniated intervertebral discs (HIVD) and the doctor. The basic data collected were used to help ensure increases in patient satisfaction and treatment.”

### 2.7 Types of outcome measurements

#### 2.7.1 Primary outcome measurement

We used a 5-point Likert scale to evaluate patient satisfaction with the doctor's explanation. Each patients answered the questions (Are you satisfied with the explanation provided by the Korean medical doctor (KMD)?, Is the KMD's explanation easy to understand?, Did the KMD's explanation cause you to feel more positively about the reliability of TKM?, Are you satisfied with the time for explanation? How much time do you estimate it took to obtain treatment on a day visit to the hospital/clinic?, Are you satisfied with the overall treatment process?). Each variable was graded according to the following scale: 1=bad, 2=not good, 3=moderate, 4=good, and 5=very good (Supplementary Table 1).

#### 2.7.2 Secondary outcomes measurement

We used a 5-point Likert scale to evaluate doctor satisfaction with the leaflet. We used a divided questionnaire for doctors who used the leaflet and doctors who did not use the leaflet for patient communication. After the explanation of the prognosis of the treatment and illness, the KMDs responded to the questionnaire to evaluate the satisfaction. The questionnaire consisted of items such as the time required for the application of the leaflet and the satisfaction and disadvantages of applying the leaflet.

Each variable was graded according to the following scale: 1=bad, 2=not good, 3=moderate, 4=good, and 5=very good (Supplementary Tables 2 and 3).

### 2.8 Statistical analysis

The statistical analysis was performed on an intention-to-treat (ITT) basis with a 95% confidence interval using multiple inputs. The ITT analysis included all randomized patients.

All analyses were performed with SAS (version 9.1.3; SAS Institute, Inc., Cary, NC) as follows: descriptive statistics were used to summarize participant characteristics; Chi-square tests were used to compare categorical data, and paired *t* tests were used to compare continuous data if participant characteristics differed among the leaflet group and the typical explanation group; and Chi-square tests (or Fisher exact tests) and paired *t* tests (or Wilcoxon signed-rank tests) were used to compare the outcomes of the leaflet group and the typical explanation group. The primary outcome was analyzed by an ITT analysis. All statistical tests were performed using a 2-sided 5% level of significance.

### 2.9 Adverse events

Any expected or unexpected adverse events were reported by the participants and practitioners at completion.

### 2.10 Ethics

This research protocol has been reviewed and approved by the institutional review board of the trial center [Jaseng KM hospital (KMJSIRB2015–39)]. Written informed consent was obtained from all study participants before enrolment in the study.

## 3 Results

### 3.1 Flow of the study

Regarding the treatment guidelines for HIVD, this cross-sectional study was conducted at the Jaseng Korean Medicine Hospital and compared a group of patients who used leaflets with a group of patients who did not use leaflets, after randomly dividing 50 shoulder pain patients into 2 groups (Table 1). These patients visited the center from September to November 2015.

**Table 1:**
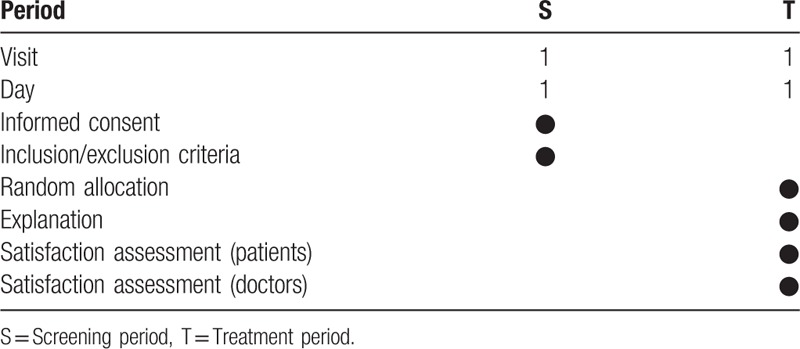
Schedule for treatment and outcome measurements.

### 3.2 Participants

The general characteristics of the participants are provided in Table 2. Among the total sample of 50 subjects, 30 (60%) were female and 20 (40%) were male. The average age of the intervention group was 41.48 ± 12.19 years, and that of the control group was 38.76 ± 11.65 years. The duration of symptoms of the intervention group was 6.60 ± 6.79 months, and that of the control group was 5.56 ± 4.81 months. In addition, the severity of symptoms was 2.88 ± 1.05 in the intervention group and 2.84 ± 0.75 in the control group.

**Table 2:**
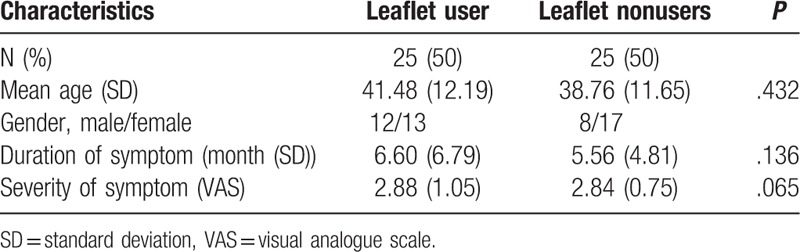
Demographic parameters of study subjects.

### 3.3 Satisfaction of the patients

The patients with HIVD answered the survey questions, which assessed their satisfaction levels based on their use of the leaflet. These data are presented in Table 3. On the basis of studies regarding the applicability of evidence-based CPGs to the treatment of HIVD, we analyzed participants’ degree of satisfaction with them and their understanding of the doctor's explanations and their resulting trustworthiness, the length of time for the medical examinations, and the overall processes of the medical treatments. The intervention patients’ satisfaction level with the overall explanation was 88%, and that of the control patients was 64%. The levels of patient satisfaction with understanding the doctors’ explanation was 92% in the leaflet group and 64% in the nonleaflet group, which showed that, compared with patient satisfaction in the nonleaflet group, patient satisfaction was 28% higher in the leaflet group. In addition, the level of the reliability with treatment was 92% in the leaflet group and 64% in the control group. However, there were no statistically significant differences between the satisfaction of the leaflet group and the nonleaflet group in terms of understanding and the level of reliability with regard to treatment. With respect to the amount of time for the medical examinations, those who used the leaflet were more likely to have visits that were 7 to 10 minutes long (44%), followed by over 10 minutes (32%) and 5 to 7 minutes (16%). In the control group, the time spent in medical examinations was 7 to 10 minutes (60%), followed by 3 to 5 minutes (20%) and over 10 minutes (12%). In addition, the satisfaction with the treatment time in the intervention group was higher than that in the control group (28%). However, there were no statistically significant differences between the leaflet group and the nonleaflet group in terms of the satisfaction with the treatment time. Regarding the frequency with which the patients visited a clinic/hospital, the highest frequency was the first time (36%) in the intervention group, followed by 5 times or more (28%), 4 times (12%), and twice (12%). The highest was 5 times or more (52%) in the control group, followed by the first time (40%). None of the patients knew the CPGs based on evidence-based medicine (EBM) in both groups.

**Table 3:**
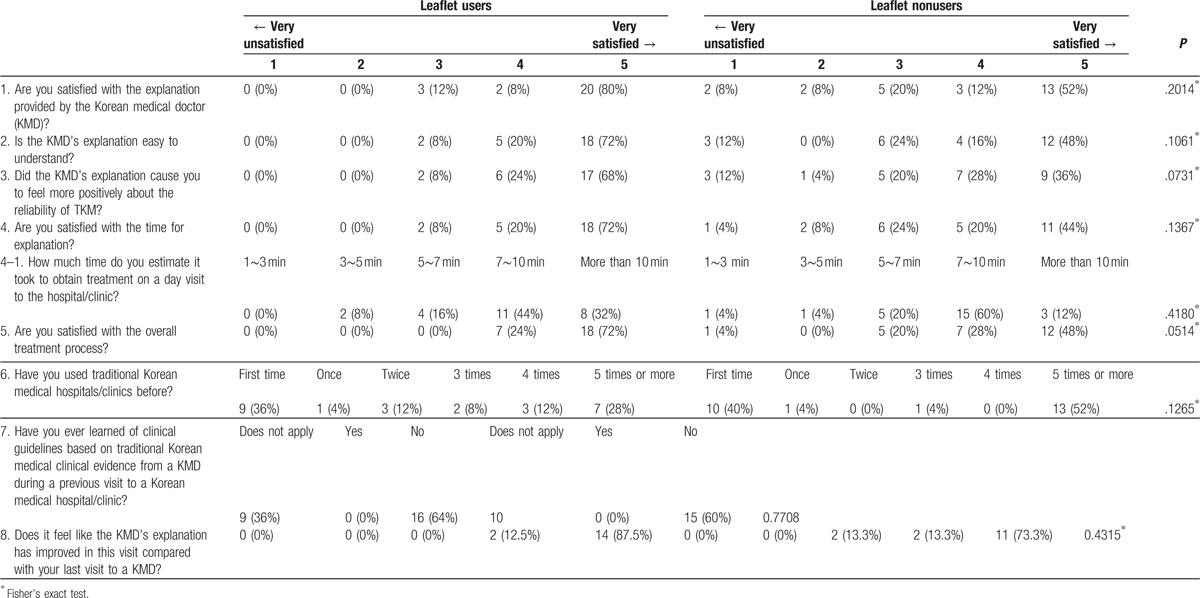
Patient satisfaction with the doctor's explanation.

### 3.4 Satisfaction of the doctors

Three TKM doctors participated in the study and were instructed to exclude as much prejudice as possible from each response and responded to the questionnaire after explaining to the patient in accordance with standard procedures. The doctors answered the questions on their satisfaction with and the necessity of using the leaflets, the data for which are presented in Tables 4 and 5. The doctors using the leaflets answered questions including satisfaction with and barriers to using the leaflet. In addition, the doctors not using the leaflets answered questions on the necessity of and barriers to using the leaflet. In the leaflet-using group, the level of doctors’ satisfaction with communicating with the patients using the leaflet was 100%. Thus, the ease of persuasion of treatment was highest (84%) and followed the improvement of the patient's understanding of the treatment (16%). However, doctors had difficulty applying the leaflet because of time constraints (48%). Their overall satisfaction with applying the leaflets was 72%, and the required time was 7 to 10 minutes. Doctors in both groups responded that the leaflets would be effective in the treatment.

**Table 4:**
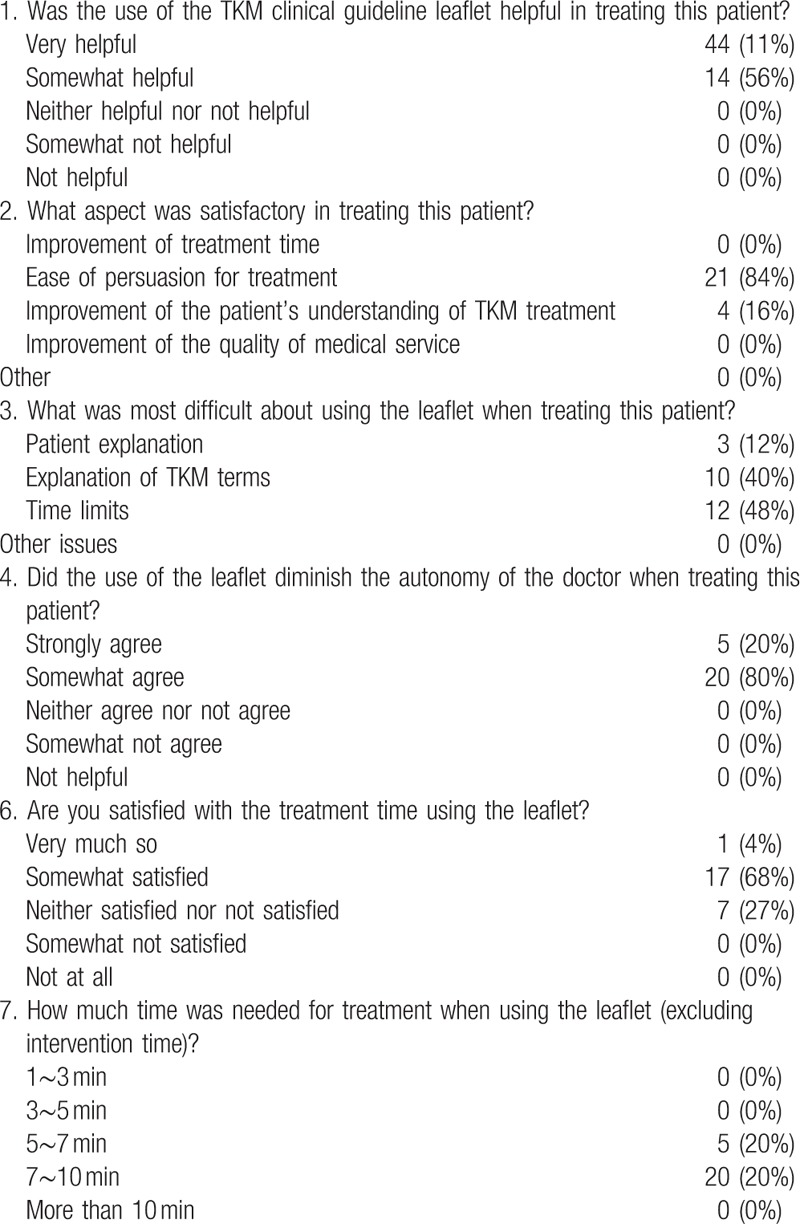
The satisfaction of clinicians with the leaflet.

**Table 5:**
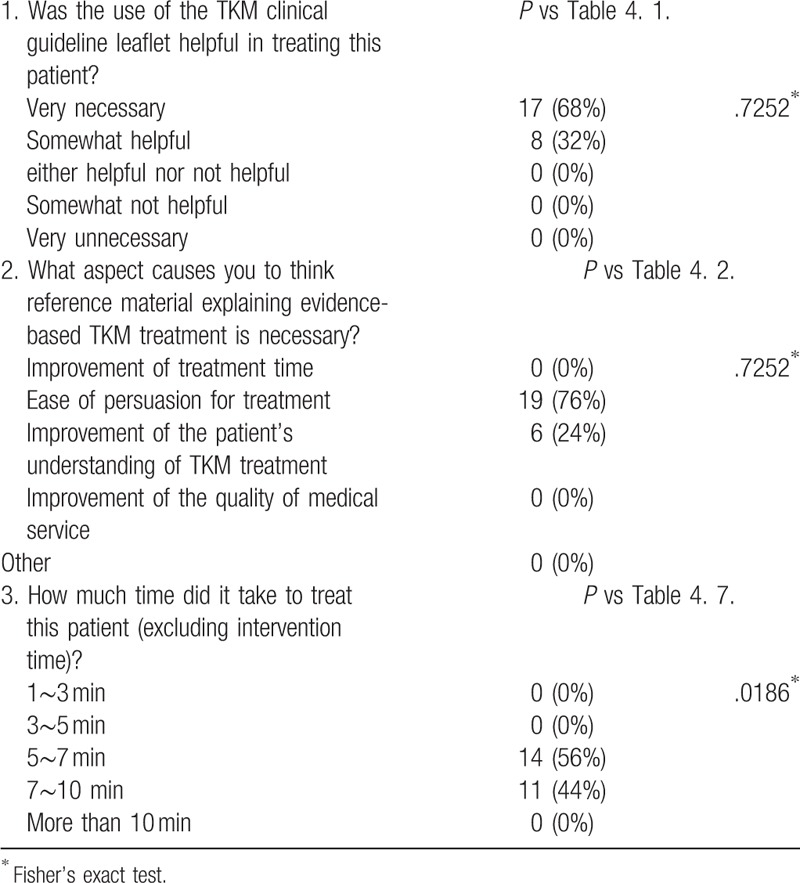
The satisfaction of clinicians without leaflet.

### 3.5 Adverse events

There were no adverse events.

## 4 Discussion

On the basis of evidence-based CPGs for TKM, which had been developed to help health care practitioners specializing in TKM to diagnose and treat diseases through a decision-making process, we used a leaflet describing evidence-based CPGs as a communication tool during the treatment of patients with HIVD.^[6]^ However, the important components of the study's results or CPGs were ignored and linked to actual clinical practice.^[15]^ Therefore, efforts are needed to disseminate the results of this study.^[16–19]^

We conducted this study with the aim of examining whether such a strategy would be helpful for increasing both patient treatment compliance and the degree of satisfaction from the perspective of both patients and health care practitioners. Thus, we examined evidence-based CPGs for the treatment of herniation of the lumbar vertebra. On the basis of studies on the applicability of evidence-based CPGs for the treatment of the herniation of lumbar vertebra, we analyzed the degree of satisfaction with and understanding of traditional Korean medical professionals’ information and the resulting trustworthiness, the length of the time for medical examinations, and the overall process of medical treatments.

Our results showed that there were no statistically significant findings between the satisfaction of the leaflet group and nonleaflet group in their understanding and the level of reliability with treatment, even though the level of patient satisfaction with understanding the doctors’ explanations was 92% in the leaflet group and 64% in the nonleaflet group. However, the level of doctors’ satisfaction with communicating with the patients using the leaflet was 100% in the user group, and almost all doctors in the nonuser group thought the leaflet would be effective in treatment (100%). Patients’ satisfaction did not differ between the 2 groups, but doctors thought that the activities in both groups would be useful in treating the patient. This finding indicates that it is much more helpful for physicians to have a tool based on the patient's explanation process. In the detailed results, the respondents answered that explanations using the leaflet would help persuade the patient (76%) and help the patient understand (24%).

There are several limitations to this study. First, this study has as open-label design. Although the outcome assessor was blinded and data analysis was performed by an independent researcher, there is still the possibility of bias. In addition, several terms of CPGs (e.g., recommendation, evidence level) may have been difficult to understand among both patients and doctors, even though it was designed to be a tool of communication.

Furthermore, we failed to show differences between the 2 groups for several reasons. First, there was insufficient statistical power of the study and an inappropriate choice of the route, dose, or frequency of administration of the intervention.^[5]^ In this study, it was unclear how many explanations should be provided to patients.

Also, severity of symptoms was below 3 of the participants who were included in this study. Considering that lumbar disc herniation is a considerably critical condition, patients with severe symptoms should have been included in this study.

Publishing the negative results of clinical trials with background information provides an opportunity to avoid possible mistakes in designing future prospective trials. It creates a basis for the modification of trial designs, which leads to new answers and new possibilities in medicine. Publishing the results is simply an act of fairness to the patients who participated in the study and experienced inconvenience and sometimes risk, with the belief that their participation will improve researchers’ understanding of which treatment works best.^[4]^ There are strong arguments for publishing the negative as well as the positive results of clinical trials because what is negative now can bring future success.

For the implementation of the CPGs, it is very important content of the leaflet itself as well as how well the guideline content is summarized, how well the patient or physician understood it, and how much the clinician agrees with the content.

Also, it is very difficult to estimate the implementation strategy based on the results of 50 patients with less severe symptoms. To apply this leaflet to other environments, such as primary care physicians or other countries, further studies with a larger number of patients and clinicians have to be done in the future.^[20]^

This approach should be followed by a variety of studies and activities to promote the development of evidence-based CPGs.

## Supplementary Material

Supplemental Digital Content
