# Supplementary material for: Evaluating the clinical application of a leaflet for clinical practice guideline in patients with lumbar herniated intervertebral discs: Randomized controlled trial
Source: Medicine (Baltimore). 2017 Dec 22;96(51):e9406. doi: 10.1097/MD.0000000000009406 (PMC5758259; doi:10.1097/MD.0000000000009406)
Supplement: Supplemental Digital Content [file medi-96-e9406-s001.doc]

Supplementary Figure 1. CPG leaflet


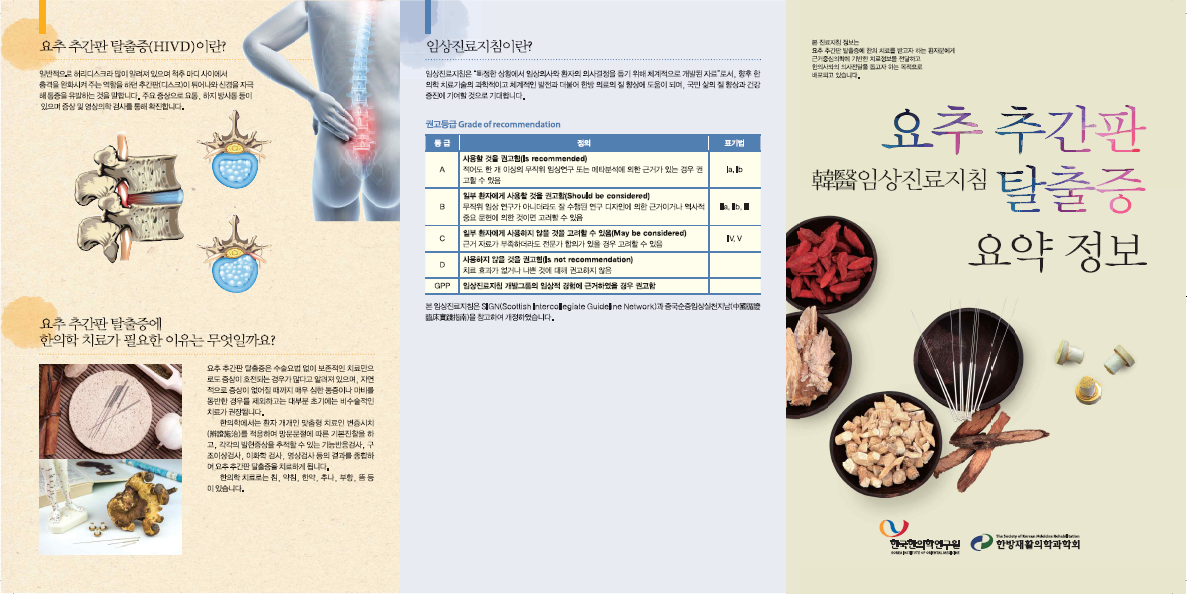


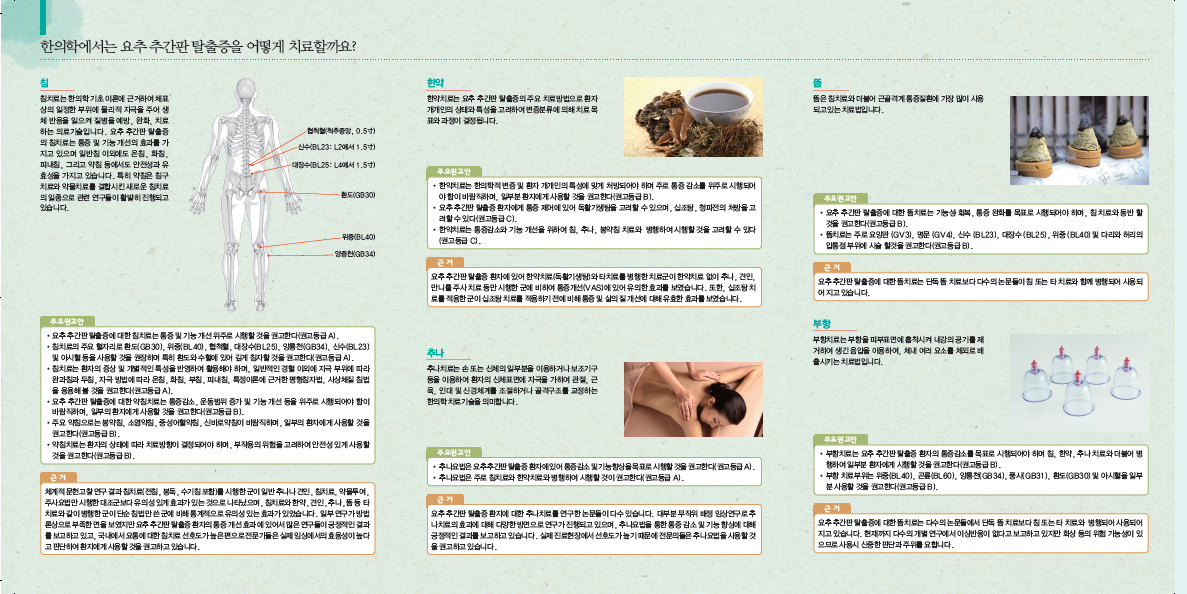


Supplementary Table 1. Survey form for HIVD patient satisfaction

| 1. Are you satisfied with the explanation provided by the Korean medical doctor (KMD)?   ← Very unsatisfied Very satisfied ⟶   | ① | ➁ | ➂ | ➃ | ➄ | | --- | --- | --- | --- | --- |   **2.** Is the KMD’s explanation easy to understand?  ← Very difficult to understand Very easy to understand ⟶   | ① | ➁ | ➂ | ➃ | ➄ | | --- | --- | --- | --- | --- |   **3.** Did the KMD’s explanation cause you to feel more positively about the reliability of TKM?  ← Did not change at all Significantly more positive ⟶   | ① | ➁ | ➂ | ➃ | ➄ | | --- | --- | --- | --- | --- |   **4.** Are you satisfied with the treatment time**?**  ← Very unsatisfied Very satisfied ⟶   | ① | ➁ | ➂ | ➃ | ➄ | | --- | --- | --- | --- | --- |   **4-1.** How much time do you estimate it took to obtain treatment on a day visit to the hospital/clinic?  ① 1-3 min.  ② 3-5 min.  ③ 5-7 min.  ④ 7-10 min.  ⑤ More than 10 min.  ⑥ Other  **5.** Are you satisfied with the overall treatment process?  ← Very unsatisfied Very satisfied ⟶   | ① | ➁ | ➂ | ➃ | ➄ | | --- | --- | --- | --- | --- |   **6.** Have you used traditional Korean medical hospitals/clinics before?  ① This is the first time  ② Once  ③ Twice  ④ Three times  ⑤ Four times  ⑥ Five times or more  -If your answer was ② ③ ④ ⑤ or ⑥, move to questions 7 and 8.  **7.** Have you ever learned of clinical guidelines based on traditional Korean medical clinical evidence from a KMD during a previous visit to a Korean medical hospital/clinic?  ① Yes  ② No  **8.** Does it feel like the KMD’s explanation has improved in this visit compared with your last visit to a KMD?  ← Not at all Very much ⟶   | ① | ➁ | ➂ | ➃ | ➄ | | --- | --- | --- | --- | --- | |
| --- | --- | --- | --- | --- | --- | --- | --- | --- | --- | --- | --- | --- | --- | --- | --- | --- | --- | --- | --- | --- | --- | --- | --- | --- | --- | --- | --- | --- | --- | --- |

HIVD: herniated intervertebral disc

**Supplementary Table 2. Survey form for HIVD medical personnel satisfaction and excellence (users)**

| 1. Was the use of the TKM clinical guideline leaflet helpful in treating this patient?   ← Not helpful Very helpful ⟶   | ① | ➁ | ➂ | ➃ | ➄ | | --- | --- | --- | --- | --- |   If your answer was ① or ②, go to Question 3.  If your answer was ③, ④, or ⑤, go to Question 2.  2. What aspect was satisfactory in treating this patient?  ① Time for explanation  ② Ease of persuasion for treatment  ③ Improvement of the patient’s understanding of TKM treatment  ④ Improvement of the quality of medical service  ⑤ Other  3. What aspect was unsatisfactory in treating this patient?  ① Problems with it being time consuming  ② Problems with persuasion for treatment  ③ Decline in the patient’s understanding of TKM treatment  ④ Decline in the quality of medical service  ⑤ Other  4. What was most difficult about using the leaflet when treating this patient?  ① Patient explanation  ② Explanation of TKM terms  ③ Visual limitations  ④ Other  5. Did the use of the leaflet diminish the autonomy of the doctor when treating this patient?  ← Strongly disagree Strongly agree ⟶   | ① | ➁ | ➂ | ➃ | ➄ | | --- | --- | --- | --- | --- |   6. Are you satisfied with the treatment time using the leaflet?  ← Not at all Very much so ⟶   | ① | ➁ | ➂ | ➃ | ➄ | | --- | --- | --- | --- | --- |   7. How much time was needed for treatment when using the leaflet (excluding intervention time)?  ① 1-3 min.  ② 3-5 min.  ③ 5-7 min.  ④ 7-10 min.  ⑤ More than 10 min.  ⑥ Other |
| --- | --- | --- | --- | --- | --- | --- | --- | --- | --- | --- | --- | --- | --- | --- | --- |

**Supplementary Table 3. Survey form for HIVD medical personnel satisfaction and excellence (non-users)**

| 1. Do you think reference material explaining evidence-based TKM treatment is necessary?   ← Very unnecessary Very necessary ⟶   | ① | ➁ | ➂ | ➃ | ➄ | | --- | --- | --- | --- | --- |   If you answered ① or ②, go to Question 3.  If you answered ③, ④, or ⑤, go to Question 2.  2. What aspect causes you to think reference material explaining evidence-based TKM treatment is necessary?  ① Time for explanation  ② Ease of persuasion for treatment  ③ Improvement of the patient’s understanding of TKM treatment  ④ Improvement of the quality of medical service  ⑤ Other  3. What aspect causes you to think reference material explaining evidence-based TKM treatment is unnecessary?  ① Problems with time consuming  ② Problems with persuasion for treatment  ③ Decline in the patient’s understanding of TKM treatment  ④ Decline in the quality of medical service  ⑤ Other  4. How much time did it take to treat this patient (excluding intervention time)?  ① 1-3 min.  ② 3-5 min.  ③ 5-7 min.  ④ 7-10 min.  ⑤ More than 10 min.  ⑥ Other |
| --- | --- | --- | --- | --- | --- |
